# Psychologization in and through the women's movement: A transnational history of the psychologization of consciousness‐raising in the German‐speaking countries and the United States

**DOI:** 10.1002/jhbs.22187

**Published:** 2022-03-03

**Authors:** Nora Ruck, Vera Luckgei, Barbara Rothmüller, Nina Franke, Emelie Rack

**Affiliations:** ^1^ Faculty of Psychology Sigmund Freud Private University Vienna Vienna Austria

**Keywords:** consciousness‐raising, history of feminist psychology, history of the women's movement, psychologization, transnational history

## Abstract

This study explores the psychologization of the women's movement by examining the activist practice of consciousness‐raising in a transnational perspective. We follow the lines along which P/psychological concepts that were appropriated and developed by North American feminist activists during the late 1960s and early 1970s traveled to the German‐speaking countries and were translated, adopted, and transformed by feminist activists in Germany and Austria. We explore both the process of psychologization as the practice traveled from the United States to German‐speaking countries and the various dimensions of psychologization: diffusion of Psy‐expert discourse beyond the borders of the psy‐disciplines, academization, individualization, and meta‐psychologization. With the latter term, we aim to capture the relationship between (feminist) P/psychology and its critique.

## 1 INTRODUCTION

Psychology is both an academic discipline and a way people feel, experience, and think about their lives. This reflexive relationship between the Psy‐disciplines and their subject matter is often described as a core tension of the discipline P/psychology (e.g., Pickren & Rutherford, 2010). Research on psychologization analyzes how the discourse of the Psy‐disciplines penetrates other social fields and/or shapes everyday feeling, experience, and action. Some scholars have argued that social movements play a particularly important role in this process (e.g., Castel et al., 1982; Malich & Balz, 2020; Tändler, 2016). Following these leads, this study analyzes psychologization processes in feminist activism in the US and the German‐speaking countries, particularly Austria, by focusing on the feminist practice of consciousness‐raising.

Research on the history of the relations between feminism and P/psychology has been able to document, especially for North America and Great Britain, a notable presence of explicitly *feminist Psychologies* that can be evidenced in sections in professional organizations, conferences, journals, textbooks, handbooks, and university courses dedicated to the advancement of feminist perspectives within Psychology (see, e.g., Brodsky, 2010; Crawford & Marecek, 1989; Dodd, 2015; Kim & Rutherford, 2015; Mednick & Urbanski, 1991; Morawski & Agronick, 1991; Rutherford, 2021; Rutherford & Granek, 2010; Rutherford et al., 2010; Tiefer, 1991; Walsh, 1985). Not least, these studies have also demonstrated the various ways in which “psychology define[s] the limits of the relationship and keep[s] feminism firmly in its place” (Crawford, 1998, p. 62). In contrast to the well‐developed historiography of feminist Psychology in North America, but especially in the United States, the history of the various relations between P/psychology and feminism have not been systematically analyzed with regard to German‐speaking countries. This void is particularly noticeable as such analyses have already been conducted for at least 14 countries or regions worldwide (see Rutherford et al., 2011). In this study, we propose that some sites of knowledge production, especially of social‐movement‐based P/psychologies (such as feminist P/psychologies), do not come readily into view when historians look primarily to the academy. When speaking of academized feminist Psychologies with a “big P,” we rely on a historiographic distinction between “big P” and “small p” psychology (Pickren & Rutherford, 2010, p. xix; Richards, 2010), that is, between the “formal, institutionalized, discipline of Psychology” (Psychology) and “the everyday psychology that has always existed as people make sense of their lives” (psychology). In distinguishing between “big P” Psychology and “little p” psychology, historians have highlighted the *reflexivity* of Psychology, that is, the fact that its subject matter constantly feedbacks into and transforms the discipline of Psychology. Richards (2010) has suggested using the convention “P/psychology” to refer to psychological knowledge and practices that encompass both Psychology and psychology. While we follow Richard's spelling, we employ the term Psychology for the discipline of Psychology and notions such as Psy‐expert knowledge or discourse to denote psychological knowledge produced not only within academic Psychology but also in the Psy‐disciplines more generally (Psychology, Psychiatry, Psychotherapy, Psychoanalysis; see McAvoy, 2014). We thus refer to academized feminist Psychologies, the form and scope of which are shaped by the Psy‐disciplines, as *feminist Psychologies*. We furthermore speak of *P/psychologies* in the plural to emphasize the heterogeneity of different feminist approaches (Sieben & Scholz, 2012, p. 14).

To analyze the history of the relations between feminism and P/psychology in the German‐speaking countries, with a particular focus on Austria, we rely on a strand of research that has studied the influence P/psychology has had on feminism since the late 1960s and the 1970s (e.g., Herman, 1995; Illouz, 2008; Schmidt, 2020). We here focus on the concept of “psychologization” that has been developed—not least from the margins of the discipline of Psychology itself—to critically assess the effects of Psychological expert discourse on society and on subjectivity (see De Vos, 2013, 2014; Malich & Balz, 2020; Mulvae & Teo, 2020; Parker, 2015). In contrast to much research on the history of feminist Psychologies, which has analyzed the effect feminism has had on Psychology, scholars interested in psychologization have focused on the other side of the equation, that is, on the impact P/psychology has had on other social fields or domains.

The relation between feminism and P/psychology has often been studied as a one‐way process of academization or psychologization: Historians have either researched the impact of feminism on the discipline of Psychology or the role of P/psychology in feminism (see Rutherford & Pettit, 2015). Illouz (2008) has argued that feminism has been implicated in psychologization processes on a larger scale. However, Illouz' general claims need to be complemented with a more detailed and situated account that attends to differences between various strands of feminism as well as to the nuances of the various P/psychologies both used and produced by feminist activists.

In this study, we explore how P/psychological concepts were appropriated and developed around the practice of consciousness‐raising by US American radical feminists during the late 1960s and early 1970s and how these concepts and practices were then translated, adopted, and transformed by feminist activists in Germany and Austria. We employ the practice of consciousness‐raising as a “lens through which to examine the interrelations of feminism and psychology” (Ruck, 2015, p. 198) and through which to understand psychologization as a process that takes place on the level of discourse within a field of practices. We first explore the uses of P/psychological notions in the earliest conceptions of consciousness‐raising developed by the radical feminist collective Redstockings in the late 1960s. In doing so, we not only attend to the swapping back and forth of concepts between feminist activists and the Psy‐disciplines (see Schmidt, 2020) but also to the interplay between psychologization and the feminist critique of the Psy‐disciplines (see Malich & Balz, 2020). Next, we analyze ways in which consciousness‐raising was psychologized already in the early by mid‐1970s, as protagonists of the Psy‐disciplines started to employ consciousness‐raising as a mental health resource for women and feminist activists, too, started to propagate more psychologized versions of the practice. The book *Feminism as Therapy* (Mander & Rush, 1974) not only serves us to demonstrate the psychologization of feminist activism but also provides us with leads to study the transnational travel of consciousness‐raising since it was translated to German already in 1976 under the title *Frauentherapie*, published in several editions, and used by activists. In thus examining how the practice of consciousness‐raising traveled from North America to the German‐speaking countries, we answer the call to indigenize the history of P/psychology (see Pickren & Rutherford, 2010; for an application of this perspective to feminist psychology Rutherford et al., 2011) and to explore the politics of location involved in the transnational travel of feminist activist knowledge (see Davis, 2007). By focusing on chapters that were added to the German translation of *Feminism as Therapy*, we aim to show that consciousness‐raising was even further psychologized in the German‐speaking countries. Finally, drawing on archival sources we explore how *Frauentherapie* was used by feminist activists in Vienna as they embarked on self‐awareness (“Selbsterfahrung”), as consciousness‐raising was commonly translated in German—the translation itself being a hint at how psychologized consciousness‐raising was as it traveled to the German‐speaking countries.

## 2 PSY‐EXPERT DISCOURSE DIFFUSES INTO US AMERICAN CONSCIOUSNESS‐RAISING

Most scholarship on the relations between feminism and P/psychology suggests that the psychologization of the women's movement was intricately connected to the radical feminist practice of consciousness‐raising (see Herman, 1995; Illouz, 2008; Rosenthal, 1984). In consciousness‐raising groups, women would typically get together in small groups to talk about their everyday experiences and they would use this sharing of experiences to analyze the social roots and conditions of their oppression. Leaders and official hierarchies were notably absent in consciousness‐raising groups.

The development of consciousness‐raising as a political method in its own right is widely attributed to the New York City‐based radical feminist collective *New York Radical Women* (NYRW), founded in 1967 by Shulamith Firestone and Pam Allen, and its later splinter group *Redstockings* (Echols, 2009; Rosenthal, 1984). While feminist activists in other places (e.g., the *Chicago Westside Group*) looked back on rather longstanding involvement in the radical movement, for example, in *Students for a Democratic Society* (SDS) or the *Student Nonviolent Coordinating Committee* (SNCC), most members of NYRW perceived themselves as being on the margins of the New Left. Some had received their political socialization in the civil rights movement rather than in the left (Echols, 2009). Though most members were on the periphery of the left, a conflict between so‐called *politicos*, that is, radicals who regarded women's liberation as a side‐branch of the New Left, and *feminists*, who supported a separate women's liberation movement in which women would organize around their own oppression, pervaded the group. Alice Echols proposed that,

women like Sarachild and Hanisch who came of age politically in the civil rights movement and who had to personally come to terms with the meaning of black power often saw the need for an autonomous women's liberation movement more quickly than women whose background was primarily in the new left (Echols, 2009, p. 73).

In their analyses of women's oppression, these radical feminist theorists would often use analogies to Black power or the Black liberation movement and compare women's oppression to the oppression of Blacks. While Redstockings and NYRW comprised of White women, many Black, Latina, Native American, and Asian women were part of most White‐dominated organizations feminist organizations, participated in many consciousness‐raising groups, and formed autonomous organizations starting at roughly the same time as White feminists (see Chávez, 2010; Taylor, 2010; Thompson, 2010).

In 1969, NYRW split into different subgroups and, after the founding of the radical feminist collective Redstockings by Ellen Willis and Shulamith Firestone, “was reduced to an umbrella group for the growing number of feminist groups” (Echols, 2009, p. 101). Redstockings was committed to the principles of radical feminism, consciousness‐raising, and action. According to Echols (2009), it was Redstockings who ultimately popularized the practice of consciousness‐raising. Redstockings founded their political program on a combination of “consciousness raising, theory, analysis and action through affiliated brigades” (Rosenthal, 1984, p. 318), conceptualizing consciousness‐raising as “both a method for arriving at the truth and a means for action and organizing” (Sarachild, 1978, p. 150). The concrete political steps, as well as the role of consciousness‐raising, can be evidenced in a program for feminist consciousness‐raising Sarachild proposed to the *First National Women's Liberation Conference*outside Chicago in 1968. Consciousness‐raising, as conceptualized by Sarachild (1970, p. 79–80), and as most likely practiced in Redstockings' early consciousness‐raising groups, can be summarized as a process that consists of four steps and that started from experience and then progressed to analysis and, finally, to political action:

- *Experience*: first, all women in a group share their experiences individually,
- *Identifying shared experiences*: second, women realize that their individual experiences are shared by other women,
- *Analysis*: third, this experience‐based realization of the collective dimension of experiences gives way to a more systematic analysis of the social origins and conditions of women's oppression,
- *Political action*: fourth, theoretical analysis provides the basis for political action on a mass level and for the changing of public consciousness.

Sarachild regarded the psychological aspects of consciousness‐raising as preliminary steps that served the purpose of changing public consciousness and, ultimately, society at large.

Sarachild's use of Psy‐expert concepts at this early stage of conceptualizing consciousness‐raising is not explicit but must be extrapolated as she did not cite any sources or name theories or scholars she drew on. We consider it unlikely that this was due to lack of academic training or ignorance, since Alice Echols has described radical feminists as mostly college educated:

It is fair to say that most early women's liberationists were college‐educated women in their mid‐to late twenties who grew up in middle‐class families. […] [M]ost of these women were unable to parlay their college degrees into good‐paying jobs. A few were pursuing careers […] [b]ut many were either working as secretaries or waitresses or working for the Movement for substance pay, or juggling both (Echols, 2009, p. 65).

Given the fact that like most other radical feminists, most NYRW and Redstockings members were college‐educated but could not, as Echols emphasizes, reap the benefits of their training, we interpret this absence of citations not as a lack of knowledge or of academic ignorance, but as an indicator that in this case, it is not the Psy‐disciplines that “define the limits of the relationship” (Crawford, 1998, p. 62) between psychology and feminism, but the social field of feminist activism.

Given that in radical feminism, self‐change was such an essential driving force in effecting social change, it is not surprising that radical feminists were especially critical of the Psy‐experts, that is, those formally involved in Psychology, psychiatry, psychotherapy, and psychoanalysis. However, though activists harshly criticized psychoanalysis (see Buhle, 1998) and other Psy‐expert discourses (see Herman, 1995), they also took up, in particular, humanistic Psychology as well as the concept of identity (Herman, 1995). While historians of the relation between psychoanalysis and feminism (e.g., Buhle, 1998) have foregrounded the critique “second wavers” launched against psychoanalysis, we further explore Herman's (1995) argument that “psychological culture” has had a considerable impact on the women's liberation movement (p. 277).

A closer look at some notions employed by Sarachild indicates that radical feminists drew rather heavily from and transformed the conceptual repertoire of psychoanalysis that was part of the psychological culture of their time. Her proposal serves to demonstrate that in thinking about consciousness‐raising, radical feminists drew on a range of Psy‐expert notions available in the cultural archive of their time (see Herman, 1995; Ruck, 2015). Psychological reflections and interventions made up a large part of the activities envisioned for the small group level, which can be evidenced in Sarachild's suggestion of activities that aimed at acknowledging so‐called “resistances to consciousness” (Peslikis, 1970, p. 81):

B. Classic forms of resisting consciousness, or: How to avoid facing the awful truth

1. Anti‐Womanism
2. Glorification of the oppressor
3. Excusing the oppressor (and feeling sorry for him)
4. False identification with the oppressor and other socially privileged groups
5. Shunning identification with one's own oppressed groups and other oppressed groups
6. Romantic fantasies, utopian thinking and other forms of confusing present reality with what one wishes reality to be
7. Thinking one has the power in the traditional role – can ‘get what one wants,' has power behind the throne, etc.
8. Belief that one has found an adequate personal solution or will be able to find one without large social changes
9. Self‐cultivation, rugged individualism, seclusion, and other forms of go‐it‐alonism
10. Self‐blame!!
11. Ultra‐militancy; and other??” (Sarachild, 1970, S. 79)

Though Sarachild does not explicitly reference psychoanalytic literature, she employs the notion of “resistance” in a vaguely psychoanalytic sense as an (unconscious) unwillingness to discuss and thus become conscious of certain topics. Resistance becomes manifest, in Sarachild's examples, in the form of identifications, idealizations, self‐deprecations, misconceptions, or reality avoidances. Furthermore, while all resistances listed above concern the psychological domain of feeling, thought, or action, some again draw on a psychoanalytic repertoire of describing psychological development more generally and so‐called defense mechanism more specifically: In her use of the concept of resistance, Sarachild varied the psychoanalytic theme of defense mechanisms developed by Anna Freud in 1936 (Freud, 1936). However, in a conceptual move that appears to combine the Marxist notion of “false consciousness” and the psychoanalytic term “identification with the aggressor” and that stripped them both of their original theoretical context, Sarachild (1970, p. 79) listed “false identification with the oppressor” as a major resistance against women's consciousness of their own oppression, thus adding more attention to systemic power relations on the one hand and focusing on objective social reality instead of individual psychological functionality on the other hand.

Sarachild was not alone among radical feminists in her use of psychoanalytic terminology to theorize and combat the psychological dimensions of oppression (see Ruck, 2015). Anne Koedt (1968) elaborated a concept of oppression via conviction and explained that oppressed groups always identified with the oppressor, themselves accepting their “inferior‐colonial‐secondary status” (p. 27) while Peslikis (1970), one of the earliest *Redstockings* and *New York Radical Women* members, conveyed the idea that there were psychological obstacles to consciousness and listed eleven thoughts that prevented consciousness. Koedt, in particular, is well known for the emphasis she put on the “psychological dimension of women's oppression” (Echols, 2009, p. 60) throughout her works.

We can see here that though radical feminists were especially critical of Psy‐expert discourse, which provided the hegemonic theories and practices of self‐change at the time, Psy‐knowledge significantly albeit implicitly infused consciousness‐raising. Castel et al. (1982) have alluded to the possibility that paradoxically, critical counter‐movements acted as special facilitators for expert knowledge—in their case psychiatry—to spread into everyday life. The insistence to “make consciousness itself the site for revolutionary engagement” unites second‐wave feminism with the therapeutic culture and the emancipatory ambitions of left social movements in the 1970s (Aubry & Travis, 2015a, p. 21; Tändler, 2016). However, radical feminism may occupy a particular place even among the social movements and counter cultures of the 1960s and 1970s because they devised such systematic (and heavily theorized) practices to bridge social change and self‐change (see Rosenthal, 1984).

We identify this spreading of Psychological or other Psy‐discipline concepts or practices beyond the borders of the disciplines (see Parker, 2015) and into ever new spheres, for example, social movements (see Malich & Balz, 2020), as the *first key aspect of psychologization* that becomes relevant for our purposes. However, we emphasize that feminist activists actively appropriated Psy‐concepts in their knowledge production, that is, in the process of using these notions they also *produced* new feminist P/psychological knowledge. We next ask how these feminist psychologies took a U‐turn and traveled back into Psychology.

## 3 CONSCIOUSNESS‐RAISING IS INCREASINGLY AND AMBIVALENTLY INDIVIDUALIZED AND ACADEMIZED

**Figure 1:**
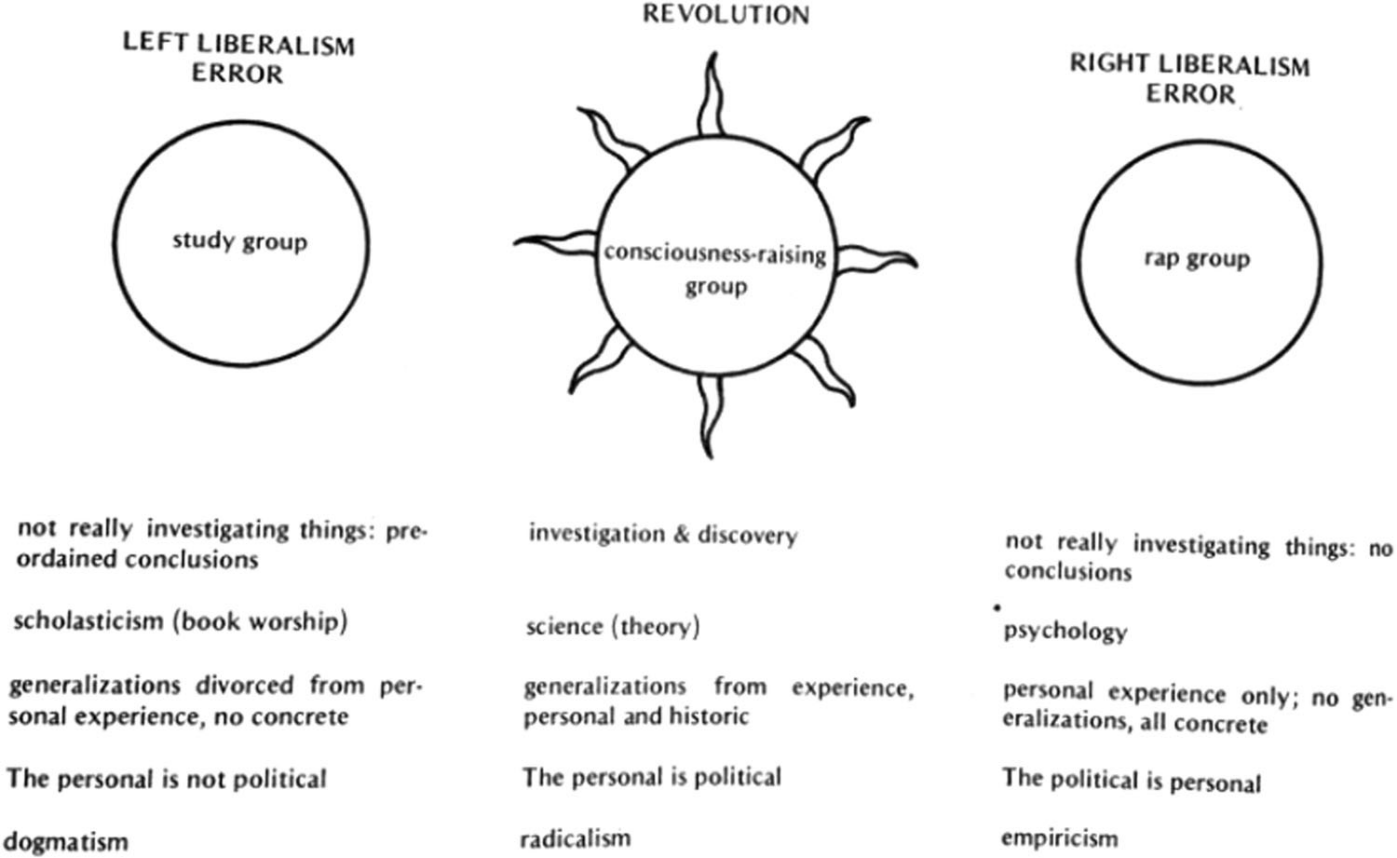
Left liberalism error—revolution—right liberalism error (Sarachild, 1978, p. 150)

While using and producing P/psychological knowledge to understand the mechanisms of both oppression and liberation, US American radical feminists were deeply critical of the Psy‐disciplines and maintained that consciousness‐raising was not therapy but an instrument for collective action. Moreover, radical feminists eyed the increasing psychologization of consciousness‐raising groups, which started only shortly after these groups gained wide‐spread and popular traction in the United States, with suspicion (see Rosenthal, 1984; Ruck, 2015). Taking stock years after drafting a program for feminist consciousness‐raising, Sarachild urged radical feminists to go back to the roots of consciousness‐raising and insisted that “the only ‘methods' of consciousness‐raising are essentially principles. They are the basic radical political principles of going to the original sources, both historic and personal, going to the people – women themselves, and going to experience for theory and strategy” (Sarachild, 1978, pp. 147–148). While Sarachild associated consciousness‐raising with revolution, she subsumed “psychology” under the heading “right liberalism error” (Figure 1).

Radical feminists had their reasons to be critical of psychologization. In North America, the practice of consciousness‐raising was going through a process of psychologization already in the early 1970s: More and more women would employ consciousness‐raising to improve their well‐being and would omit the more political steps of process. This *individualization* of practices once intended to collectivize and politicize is another key aspect of psychologization—and a main target of the critique of psychologization (see De Vos, 2014). Particularly, activists and left intellectuals suspected that psychologization isolated the individual from collective communities and prevented them from taking radical action (see Aubry & Travis, 2015b). Autonomy, self‐actualization, happiness, and the obligation to individual choice are viewed by the literature on therapeutic culture to be the core characteristics of an individualization process that was easily absorbed by a capitalist logic of accumulation (see Ahmed, 2010; Illouz, 2008; Rose, 1989). By contrast, critiques from the other side of the political spectrum, mostly conservative agents, objected to the therapeutic distraction of individuals from their civic duties (see Aubry & Travis, 2015b).

Consciousness‐raising increasingly and explicitly fed back into expert discourse and practices within the Psy‐disciplines. In the United States, for example, discussions about the relevance of consciousness‐raising for psychotherapy reached some of the official Psychological publications in the early to mid‐1970s (see Ruck, 2015). The journal *Psychotherapy* of the *American Psychological Association*'s Division 29 *Society for the Advancement of Psychotherapy*, for example, published articles on consciousness‐raising rather regularly from 1973 onwards (e.g., Brodsky, 1973; Ellis & Nichols, 1979; Fodor, 1974; Gerson, 1974; Johnson, 1976; Kravetz, 1976; Lieberman & Bond, 1976; Salwen, 1975). Psychologists also began to systematically study the effects of consciousness‐raising (e.g., Follingstad et al., 1977) and proposed consciousness‐raising as an alternative resource for promoting women's mental health. The Psychologists and therapists who dealt with consciousness‐raising also emphasized the differences, however: In contrast to group therapy, consciousness‐raising assumed, for example, that the problems of the individual were related to social problems (Brodsky, 1973). In addition, consciousness‐raising aimed to establish egalitarian and mutual relationships, while in therapy the roles of therapist and patient were not interchangeable (Kravetz, 1976).

This development points to another aspect of the psychologization of the women's movement that we refer to as *academization*. Most radical feminists associated with the inception of consciousness‐raising were college‐educated, some had participated in student‐dominated organizations of the New Left, and most were familiar with the theories these radicals drew on (see Echols, 2009). What is more, the practice of consciousness‐raising increasingly traveled from the social field of activism, in which it had been developed, to another social field where it was transformed according to the rules of the new field; that is, consciousness‐raising traveled from radical feminist activism to the Psy‐disciplines, where it was elaborated into explicitly feminist therapies or other, mostly group‐based approaches.

However, feminists themselves further developed consciousness‐raising in a rather psychologized fashion, too. In 1974, US West Coast feminists Anne Kent Rush and Anica Vesel Mander published *Feminism as Therapy*, a book feminist therapist Miriam Greenspan remembered as one of “only two full‐length books I knew of that directly addressed the subject” of “therapy from a feminist perspective” (Greenspan, 2017, p. 339) in the 1970s, the other being *Notes of a Feminist Therapist* by Elizabeth Friar Williams published in 1976. In contrast to radical feminists of the East Coast, who had incepted the practice of consciousness‐raising, both Mander and Rush were part of the counter‐culture scene around San Francisco.

Anica Mander Vesel, born in Yugoslavia, fled the National Socialists with her family during the Second World War and arrived in the USA in 1949 at the age of seven. By the time she co‐authored *Feminism as Therapy*, she had been expelled as a lecturer in linguistics from *San Francisco University* for joining Black students in a campus strike (*Los Angeles Times*, 2002). She subsequently led the feminist studies program at *Antioch College West* in San Francisco (see Grenn‐Scott, 2000). Anne Kent Rush, born in the United States in 1945, moved to Berkeley, California, in 1969. She learned massage techniques at the *Esalen Institute* in Big Sur, California, and verbal Gestalt techniques at the Gestalt Institute of San Francisco (Rush, 1978, p. 15). Both locations were important centers for innovative forms of (psycho‐)therapy. When *Feminism as Therapy* was released, Rush had just written *Getting Clear: Body Work for Women* (1973), which marked the beginning of her brisk publication activity in the following decades. Most of her writing focused on body work like massage and yoga.

In the following, we explore the different layers of psychologization in *Feminism as Therapy*, especially with regard to how the authors dealt with Psy‐expert discourse, in more detail. With the exception of the introduction, most chapters in *Feminism as Therapy* were written by one of the two authors. In their shared introduction, Mander and Rush all but equated feminism with therapy: “feminism can and does function as therapy” (1974, p. 3).^1^ Rush furthermore compared therapy with consciousness‐raising: “I prefer to view therapy as a consciousness‐raising process. That is, our getting together to figure out ways we can improve our relating and make some advances in human interaction” (Rush, 1974a, p. 37). Rush explained that the integration of the personal and the political allowed feminism to work as therapy, as feminism could only function as therapy because it included the political (Rush, 1974a, p. 57).

Mander and Rush were critical of contemporary forms of therapy, of psychoanalysis, and of various aspects that we have identified as features of psychologization, thus themselves partaking in a critique of psychologization. Furthermore, like many other feminists, they were at least suspicious of traditional academic practices. Both authors shared some of their employment histories in the introduction: Mander had taught languages apparently at different universities for fifteen years and had been fired “on the grounds that my qualifications did not meet the criteria set up by white men” (Mander & Rush, 1974, p. 4). Rush had quit her job at a publishing house “when they told me i [sic] couldn't have more pay or be taken seriously because i was a woman” (Mander & Rush, 1974, p. 5). Both expressed suspicion with academic institutions and practices at various points:

Those of us who have been *well‐educated*, that is, brainwashed with male concepts, know a lot of theories: we know that the facts need to be documented, we know that logic can guide us toward acceptable conclusions and we know that if we want to make it in the world, we will not cry, we will not lose our temper and we will not interrupt. As i write this sentence i *know* that i *should* be documenting what i am saying, that i *should* cite sources, and that i *should* have footnotes and statistics. But there is another knowledge operating – one that has replaced the old: the knowledge that i have gained from other women. The only theories that work for me are those that match up with my feelings. […] We know that it is our passion, our anger and our tears (our *hysteria*, as it is commonly called) that have brought us to this point: the one of action, of assuming responsibility, of political consciousness. (Mander & Rush, 1974, p. 21)

Mander and Rush charged psychoanalysis with being particularly implicated in women's oppression. Rush (1974a), however, considered Freud “confused” (p. 39)^2^ and made a distinction between “Freud's original outlook and writings” and “the social mythology that has become ‘Freudian psychology' in our culture, as it is practiced in the minds of families, advertisers, industrial consultants, psychiatrists and psychologists day to day.” It was the latter she was most critical of. Based on this differentiation, Rush engaged with Freud's original writings, warning, however, that most contemporary approaches to his theories were uncontextualized, and that much of what passed as Freudian psychology was based on secondary instead of primary sources. It was mostly this strand of psychoanalytic therapy that she blamed for having as its goal the adaptation of individuals to their environment. Rush asserted that in his original writings, Freud “made the unfortunate mistake of confusing power with sex” (p. 40). She redefined Freud's concept of “penis envy,” arguing that women's desire to belong to the opposite sex was based on family and social hierarchy and not on “natural” female “weakness.” Rush then went on to engage with other psychoanalytic or therapeutic schools in a critical but nonetheless affirmative way, that is, with Emma and Carl Jung, and Wilhelm Reich. She regarded Gestalt psychological methods as helpful for feminist therapy. However, she criticized Gestalt therapy for overemphasizing individual responsibility, which in her view made it difficult for women to analyze the relationship between their individual problems and social systems as well as to locate responsibility for their problems within the system instead of themselves.

Feminist therapy strove to overcome the contradictions and shortcomings of traditional therapies. Mander and Rush's concrete suggestions relied mostly on body work and body‐based therapy techniques (Rush, 1974b). Some concrete basic techniques were to perceive closely into the body when one experienced intense feelings, and attending to and altering one's breath. Other more specific techniques were grouped into the themes “sex roles and self‐image,” “trust,” “responsibility,” “anger,” “mothers and daughters,” “female spirituality,” and “play.”

*Feminism as Therapy* is yet another example of how Psy‐expert discourse was taken up in feminist discourse through an intricate interrelation of critique and adaptation. However, it also illustrates that the varied ways in which feminist activists dealt with Psychological and other Psy‐expert sources. As we noted above, we had to extrapolate Sarachilds' early use of Psy‐expert notions since she did not cite any authors or texts. Mander and Rush, by contrast, displayed that they were knowledgeable of academic practices and standards of dealing with sources and intentionally denounced them. While Mander and Rush refused to adhere to academic styles of citation that were already in place at the time, they did name psychotherapeutic schools and their founders such as Sigmund Freud, Emma and Carl Jung, or Wilhelm Reich. Rush (1974a) even complained that many proponents of Freudian psychoanalysis failed to engage with the original sources. Though the authors expressed their critique of “traditional” academization, we argue that they nevertheless put forward the process of academization of feminist activist knowledge production by advancing academic practices such as explicitly engaging with academic primary sources and further developing theoretical notions by other authors.

## 4 FROM *FEMINISM AS THERAPY* To *FRAUENTHERAPIE*: A PSYCHOLOGIZED VERSION OF CONSCIOUSNESS‐RAISING IS TRANSLATED INTO GERMAN

Consciousness‐raising traveled to other countries at a time when the practice was already widely but controversially psychologized in the United States. In fact, consciousness‐raising psychologized so rapidly in its context of origin that when the practice traveled to Canada in the early 1970s, it was used in a rather psychologized fashion early on (see Ruck, 2015). However, the resources available to women in other countries that started engaging with consciousness‐raising varied. For example, resources at hand for Canadian women who intended to start a consciousness‐raising group were largely activist: A consciousness‐raising kit made available by the Canadian women activist center (*Toronto Women's Place*) that had started offering consciousness‐raising groups in 1972, for example, contained mostly radical feminist leaflets and texts.

By contrast, as we will show, resources available to German‐speaking women who started engaging with consciousness‐raising in the early 1970s were already more professionalized or academized, and certainly psychologized. Two years after the publication of *Feminism as Therapy*, a German translation initiated by the *Psychologinnengruppe München*, a group of female Psychologists from Munich, was published in 1976 under the title *Frauentherapie: Frauenbewegung als heilende Energie* (*Women's Therapy: Women's Movement as Healing Energy*).^3^ Noticeably, the label “feminism” and the equation of feminism with therapy signaled in the English title did not make it to the German translation.^4^ While the American original was co‐published by the major publisher *Random House* with a one‐time circulation of 26,500 books (Mander & Rush, 1974, title page), the German translation was released by *Frauenoffensive*, the first women's book publishing house in Germany. *Frauenoffensive* was a small publishing house which was able to become autonomous from the leftist publisher *Trikont‐Verlag* in 1976 because one of its first books turned out to be an unexpected success (Hermanns, 2020). Between 1976 and 1981, *Frauentherapie* was published there in six editions: In 1979 alone, the third and fourth edition were released. The publishing group expanded their average circulation range between 3000 and 5000 copies (Zellmer, 2011, p. 194). The autonomous publishing group was based in Munich and closely connected to Munich's lively feminist subculture.

In 1978 *Emma*, a nationally known German feminist magazine, featured an interview with the publishers at the new *Frauenoffensive Verlag*. In this article, the publishers elaborated why Anglo‐American books had been particularly relevant to their program: Germany lacked what they called the “great tradition of Anglo‐Saxon women's writing” (Münch, 1978, p. 12). Many texts submitted by German‐speaking female authors were rejected, mostly because the publishers missed innovative ideas or because the texts were produced for academia and the publishers therefore considered their style as too scientific for a broader audience. One of the principles expressed by the publishers was that they did not want to publish material that would prompt women to adapt to the status quo rather than creating new ways of being. In contrast, they found what they were looking for in American feminist literature: “Through reading foreign, especially American texts, we were confronted with feminist poems, novels, short stories. This form of expression came into play within the framework of a more developed feminist approach – starting from ourselves” (Frauenoffensive Journal, 1976, Nr.4, p. 56). Beyond this, the selection of texts was certainly also influenced by the fact that the publishers responsible for the “foreign editorial” had studied American Studies and English (Münch, 1978). Finally, the publishers stated that their publishing decisions were intricately connected to their own feminist development: In the beginning, their program mostly included texts about the oppression of women, then, on women's relationship to their bodies and, last, psychotherapy for women. Notably, the only other book in that genre mentioned in the *Emma* article was *Getting Clear* by Anne Kent Rush.

The publishers at *Frauenoffensive* wanted to publish new, innovative texts and preferred to translate rather unknown books, thus aiming to distance themselves from their male colleagues and their publishing principles:

It is true that men's publishing houses are also willing to publish women's literature, especially in the Year of the Woman. […] The principle of men's publishing houses, however, is to pick out and market for themselves what is already finished, already “successful” and, so to speak, established. We, however, want something different: we create a “protective space” in order to develop and practice new models of thought and action. (Frauenoffensive, 1975).

*Feminism as Therapy* thus fit neatly into the program of *Frauenoffensive*: The authors expressed their ideas from a personal standpoint, deliberately ignored academic rules, and conveyed complex content in simple words. The fact that this resulted in some flattening of theoretical content is noted in the preface but accepted in view of the ultimate goals (psychological emancipation of women).

The *Psychologinnengruppe München*, who wrote the foreword to *Feminism as Therapy* in the German translation of the book, consisted of Helga Bilden, Annemaire Blessing, Luise Lehmann, Hanna Permien, and Cornelia Schwartz (Münchner Psychologinnengruppe, 1976, p. 11). The Munich Psychologists emphasized their conviction that the book should be translated and discussed “at this moment of time in the women's movement, where the need for personal change has moved to the fore for many women and where women are seeking inspiration and guidance to effect this change” (p. 5). 1976—“this moment in time”—was the year in which, according to the Munich group, “psychotherapeutic women self‐help groups” (Psychologinnengruppe München, 1978, p. 249) were founded for the first time in Germany. In their self‐description they stated that they were “the participants in the first generation of Munich self‐help groups, [who] often knew each other from earlier groups or work contexts, were mostly psychologists or studied social sciences, had generally similar attitudes toward issues of the women's movement, and were all between 25 and 40 years old” (Psychologinnengruppe München, 1978, p. 252).

According to the Munich group, these developments in Germany were inspired and initiated by American feminists who came to Europe. On lecture tours they reported about the “foundation of women's health centers and the development of new treatments” and the new ideas were “enthusiastically embraced” by German feminists (Psychologinnengruppe München, 1978, p. 245). Outlining their motivation only somewhat later, the Munich Psychologists described self‐awareness groups as precursors to their approach because these groups made it possible for the first time to understand women's suffering and fear “not as personal failure but a meaningful reaction to their objective situation” (Psychologinnengruppe München, 1978, p. 247). Though self‐awareness groups were an important inspiration, the Munich group clarified that in this setting, there was no space to work on individual psychological problems. Instead, they aimed to create a psychotherapeutic group in the manner of medical self‐help groups and thereby positioned their project in between the political and the medical approach.

At different points, the Munich Psychologists expressed their critique of *Feminism as Therapy* in their foreword to the German translation. In particular, they were ambivalent about the definition of therapy proposed in *Feminism as Therapy*: While Rush considered therapy as a process of raising women's consciousness and changing their interactions, the Munich Psychologists emphasized the collective and political aspect more strongly:

After reading the book, we think that ‘therapy' here, when referring to the therapeutic moments of the women's movement (or feminism), is to be understood rather as: all processes that serve the goal of liberating and developing the suppressed subjective potential of women collectively and individually, so that they change oppressive conditions, reduce gender roles. (Psychologinnengruppe, 1976, p. 7).

In this quote, changing oppressive conditions is all but equated with changing gender roles. This somewhat psychologizing tendency that is only implied in the introduction is more pronounced in the two more practical final chapters *Women's Talking Groups* (“Frauengesprächsgruppen”; Wagner, 1976) and *Suggestions for Further Activities* (“Hinweise zum Weitermachen”; Münchner Psychologinnengruppe, 1976) that were added in the German edition. Not least because the book was used as a resource by Viennese self‐awareness groups, we consider these chapters as especially illuminating with respect to the ways in which consciousness‐raising traveled to the German‐speaking countries. In *Suggestions for Further Activitie*s, the Munich Psychologists defined CR or talking groups the following way: “These groups attempt to create alternative forms of communication and the experience that the situations and challenges of women are about the same” (Münchner Psychologinnengruppe, 1976, p. 142).

Psychologist Wagner (1976) who wrote the appendix on women's talking groups in the German translation of *Feminism as Therapy*, played a pivotal role in the spreading of CR groups in Germany. Wagner, born in 1944 near the city of Dresden, Germany, studied pedagogy and psychology in Bonn and Hamburg, Germany from 1963 to 1967 (Metz‐Göckel, 1979, p. 208). Afterward, she went to the United States and completed her M.A. in psychology at Southern Illinois University and earned her doctorate at the University of Michigan in 1971. It was in Ann Arbor where she first became active in the women's movement: Wagner participated in two CR groups there and became a member of the *Association for Women in Psychology* (Metz‐Göckel, 1979, p. 208). Back in Germany, she first held a position as lecturer at the Pedagogical University in Reutlingen, Germany and in 1974 became professor for Psychology there. In 1975 she was involved in the founding of a women's center in Reutlingen where she conducted assertiveness trainings for women.

A closer look at a selection of her works allows us to understand how she helped to spread CR groups in Germany. In 1972 Wagner first initiated what she called *Frauengesprächsgruppen* (Metz‐Göckel, 1979, p. 208), (women talking groups) at the Volkshochschulen (folk high schools or community colleges) in Reutlingen. One year later Wagner's text “Awareness changes through emancipation discussion groups” (Bewusstseinsveränderungen durch Emanzipations‐Gesprächsgruppen) was published in a book on the social‐psychological aspects of misogyny (Schmidt et al., 1973). Wagner introduced—by referring to the New York *Redstockings*—the origin of CR groups in the United States, explained what their purpose was and how the groups should work and be structured. According to Wagner, CR groups have a “politicizing effect in the basic sense” (Wagner, 1973, p. 154), further clarifying politicization as “the insight that what is always seen as a symptom of one's own insecurity or frustration is so regularly found in other women that it must be seen as socially induced” (Wagner, 1973, p. 154). In Wagner's reasoning, however, the political dimension of CR takes a backseat. Wagner points out that “emancipation‐talking‐groups” (Wagner, 1973, p. 155) can help to develop self‐confidence, free “psychic strength which until then had been buried under depression and feelings of inferiority” (Wagner, 1973, p. 155) and therefore the groups can also have a therapeutic effect. According to Wagner, CR groups were largely unknown in Germany when she wrote this article in spring 1973 (Wagner, 1973, p. 145).

One year later, the conservative but wide‐ranging women's magazine *Brigitte* published an article on so‐called rap‐groups (Leeb, 1974). The author visited one rap‐ or CR‐Group in Reutlingen founded on the initiative of Angelika C. Wagner, and also cited and interviewed the professor herself. In the study, Wagner explained the concept and the rules of a CR‐group in a basic way. To create a lively insight for the readers, five group participants shared their experiences. Each of them related an aspect of the development initiated by their CR‐group (accepting one's own body, learning to talk in front of groups, being more assertive and changes in heterosexual relationships). One woman stated that she missed “the political background in the group. It is not enough that one emancipates and creates a free space for oneself, but leaves the whole crappiness [Beschissenheit] of this society as it is” (Leeb, 1974, p. 92). Compared to the general tone of the conservative German magazine of the 1970s, where appearance, child‐care, cooking, housekeeping, and so forth were central topics, this quote is rather provocative. Feminist collectives also published texts on CR groups in the early 1970s, for example, the *Frankfurter Weiberrat* in 1971 (Flügge, 1998) but their activist publication organs had a significantly smaller range of impact than *Brigitte*. In an interview, educationalist Sabine Hering recalled that she met Wagner in 1973 or 1974 and participated in one of her by then already “famous consciousness‐raising groups that she had brought with her from America at that time” (Bock, 2015, p. 90).

By the time Wagner 97 wrote the appendix for *Frauentherapie*, she was thus already known for her theoretical and practical work on talking groups. The appendix is rather similar to her text from 1973 but written in a less academic style. Again, Wagner explained that the idea originated in the United States and she also called them “chatting groups” (*Quatschgruppen*), “self‐awareness groups” (*Selbsterfahrungsgruppen*), and “CR‐Gruppen” (*cr‐groups*)—without, however, clarifying what CR stood for. After summarizing the characteristics of women's talking groups, Wagner commented on their effects: Most importantly, women experienced that what they had thought of as their individual problem was in fact shared by other women, which often came with great relief. Furthermore, the groups expanded women's horizons and helped to overcome competition as well as to develop feelings of solidarity. Wagner explained that women's talking groups were not therapy groups: “In therapy, problems are explained and treated as results of the *individual* life story (childhood). In talking groups, experiences are looked at in relation to the *general* situation of women” (Bock, 2015, p. 129). Questions suggested by Wagner included, for example, “How did our parents relate to us girls? Were we raised as ‘girls'? What feelings did this evoke in us at the time” (Wagner, 1976, p. 139). Similar questions were formulated for themes such as adolescence, school education and profession, sexual fantasies and sex, marriage, power and success, hopes, experiences with discrimination, and relationships with older women.

Altogether, Wagner's German‐speaking texts on CR groups suggest to us that she promoted a rather psychologized version of CR groups. After all, when the first ‘self‐awareness groups' began to be founded in Germany in the early 1970s and, a bit later, in Austria, the practice of consciousness‐raising had already been heavily but controversially psychologized in North America. The common translation of consciousness‐raising groups as "Selbsterfahrungsgruppen” (literally “self‐experience groups” or “self‐awareness groups”) may serve as another indicator that consciousness‐raising developed in the German‐speaking countries in an already somewhat psychologized fashion from the beginning, not only but also because the resources translated into German, for example, *Feminism as Therapy*, provided a psychologized reading of consciousness‐raising.

Ironically, some of the Psy‐resources critiqued in *Feminism as Therapy*/*Frauentherapie*, particularly the psychoanalytic authors and approaches, had their origins in the German‐speaking countries: Austria (Sigmund Freud, Wilhelm Reich), Switzerland (Emma and Carl Jung), and Germany (Laura and Fritz Perls and other Gestalt Psychologists). In Germany and Austria, discourses about psychoanalysis and Gestalt Psychology were violently interrupted in the 1930s by the Nazi dictatorship. While some strands of psychoanalysis flourished in Nazi Germany under patronage of Matthias Heinrich Göring, a cousin of Hermann Göring's (one of the highest ranking national socialist politicians), Jewish psychoanalysts, in particular, were systematically persecuted or murdered and only some managed to escape from Nazi Germany: Freud lived his last years in the United Kingdom, while Reich and the Pearls fled to the United States (Cocks & Albion, 1983). In the course of the second women's movement, feminist literature from the United States was imported to Germany and that also included feminist receptions and critiques of psychoanalysis. These authors thus made their way to feminist activists in the German‐speaking countries through the detour of their reception by the US American feminists. Through the networks between German‐speaking feminists, which were especially strong between Munich (Germany) and Vienna (Austria), *Frauentherapie* then traveled from Germany to Austria. As we will see in the next section, the book was among the resources Viennese activists used as they first took up the practice of consciousness‐raising, or rather “self‐awareness.” Upon its arrival in Austria, consciousness‐raising took another U‐turn through the bottleneck of psychologization and traveled back to activism in a fashion that had integrated various therapeutic schools and principles.

## 5 “SELF‐HELP IN THE PSYCHOLOGICAL FIELD”: CONSCIOUSNESS‐RAISING ARRIVES IN VIENNA

In Austria, the founding of the *Aktion Unabhängiger Frauen* (Initiative of Autonomous Women; AUF) in Vienna in 1972, marked the inception of the second wave women's movement in Vienna (see Krondorfer & Grammel, 2010). Brigitte Geiger and Hanna Hacker's book *Donauwalzer Damenwahl* continues to represent the most comprehensive history of the first two decades of the Austrian second wave women's movement to date, especially with regard to the autonomous women's movement AUF (see Mayer, 2018). According to Geiger and Hacker (1989), the founders of AUF were a rather homogenous group of mostly young Austrian women, the majority of whom studied at university and leaned towards the social sciences. Originally rather idealistic and motivated to get down to work, many working groups within AUF were unable to realize their goals because a clear through‐line was missing, opinions were too diverse, and there was a high turnover due to volunteerism and a lack of friendly ties, as was the case, for example, with the working group on education (“Arbeitskreis Erziehung”) (see Geiger & Hacker, 1988, p. 96). Over time, the growing need of AUF women to secure their own existence necessitated increasingly bureaucratic and professionalized structures, which inevitably contradicted their initial political ideals and goals. Some AUF women regarded these new structures as a “capitalization of relationships”. Such ambivalences led to diffusion and “parcellization” within the AUF (p. 151) and some women turned to political parties. After 1977/1978, the range of university groups diversified, while there were hardly any AUF groups of their own, a development Geiger and Hacker interpreted as a sign of “decentralization” (p. 285). White antiracist activism started in the 1980s (Mayer, 2018), when in the wake of the project movement, self‐organized migrant women's groups started putting migrant women's issues front and center.

From the beginning, AUF included a number of so‐called “communication groups” or “self‐awareness groups”. Already in 1973, there were at least ten parallel groups of women in Vienna, who met regularly to talk about their experiences in the group (see Geiger & Hacker, 1989). Communication groups were set up to follow the principles of consciousness‐raising and to integrate newcomers. Starting in 1975, there was an increasing focus on a working‐group devoted to feminist therapy and other therapeutic groups. Geiger and Hacker called what we frame as an increasing psychologization of AUF a “turn to the ideology of self‐awareness” in AUF (1989, p. 40; German original). In 1976, AUF renamed themselves *Women's Centre* (Frauenzentrum). In the same year, there seem to have been 300 to 500 self‐awareness groups in the Federal Republic of Germany (Geiger & Hacker, 1989). In AUF/Women's Centre, there were five communication groups and four self‐awareness groups in 1976.

Somewhat later than the turn to therapy that occurred in AUF/Women's Centre, self‐awareness became associated with processes of institutionalization and professionalization. Geiger and Hacker call this process an increasing “professionalization of self‐awareness” (p. 119). In 1977, women who were active at Vienna's *Volkshochschulen* (folk high schools or community colleges),^4^ founded the *Frauen Foren* (Women's Forums) and increasingly, self‐awareness would be practiced there (see Geiger & Hacker, 1989). Volkshochschulen traditionally supported emancipatory politics, were an important educational resource for women, and played a significant part in the educational structures of the second wave (see Bundeskanzleramt Österreich, 1985; Petrasch, 2007). At the Viennese *Urania*, the Volkshochschule of the first district of Vienna, a women's forum was established in the autumn of 1977 and a self‐awareness group was launched. Gerlinde Schilcher, an employee at Urania, used the institutional resources accessible there to form a women's discussion group. Due to considerable demand, the Women's Forum Urania gradually expanded and (former) group participants began to lead other women's groups themselves.

The Women's Forum appealed mostly to women in their mid‐ to late‐30s with professional experience who were married or divorced and often had children (see Geiger & Hacker, 1989, p. 119). According to Geiger and Hacker, Women's Forum considered itself as part of the autonomous women's movement. There was only marginal exchange with the Vienna Women's Centre (former AUF), though. Women's Forum initiators knew AUF/Women's Centre but did not feel intimately connected to it; Women's Centre, in turn, followed Women's Forum with solidarity and distance but assumed a different target group (mothers, housewives, and working women) or suspected that Women's Forum promoted “career orientation.” The relationship between the autonomous women's movement and the Women's Forum was soon strained by ideologized attributions and projections as well as conflicts. While women of the AUF experienced their joint work in a positive sense as “official” and “political” (Geiger & Hacker, 1988, p. 45) and classified the emerging debates and analyses as “lively” and relevant, women at the Women's Forum perceived the culture of discussion as hierarchical and ideological. From “eternal discussions of principles” both sides parted “in exhaustion” (p. 195), as former AUF activists reported in interviews with Geiger and Hacker.

Notwithstanding the differences and sometimes conflicts between the different collectives that hosted self‐awareness groups, the feminist approach to self‐awareness was increasingly anchored in Volkshochschulen (Geiger & Hacker, 1989, p. 120). According to Geiger and Hacker, participants—especially the more activist oriented ones—experienced the concrete practice of self‐awareness groups as ambivalent, because gradually the political objectives and character of self‐awareness groups took a back seat to psychological aspects and individual self‐discovery. For this reason, the autonomous women's movement often used these groups as an entry aid of limited duration for newly recruited women.

Drawing on both archival records of AUF publications and on interviews Geiger and Hacker (1988) conducted with former AUF activists, we now want to look more closely at the earliest groups founded in AUF, some of the ways in which they practiced self‐awareness, and the conceptual resources they drew on. The first communication group, founded within AUF at the beginning of 1973, formulated the need of its members to discuss problems, to present themselves, and to analyze problems on a more abstract level (see Geiger & Hacker, 1989). The women hoped that this would lead to a better understanding of themselves and other women. The group entered the process rather unsystematically first and, according to Geiger and Hacker, then decided to employ a questionnaire developed by a US American consciousness‐raising group.^5^ However, the suicide of a member in one of AUF's communication groups must have been a drastic experience that prompted the group to employ more systematic methods and guidelines for self‐awareness groups. As one former member recounted in an interview:

She always talked about such terrible conditions in her relationships and the problem with her mother. But she always talked so casually and always used technical terms, and somehow it didn't get through to me, to us. […] Well, in short, in December, at Christmas 75, the woman killed herself […] The group then broke up behind the case, because we had more or less reached the limits. To the limits of ‐ well, it just wasn't a therapy group. (Quoted in Geiger & Hacker, 1989, p. 122)

In 1976, two women initiated a working group on feminist therapy in AUF. From the winter of 1976 to the summer of 1978, the working group *Feminist Therapy* was announced in *AUF—eine Frauenzeitschrift* (*AUF*—*a women's magazine*), which served as one of the main communication platforms of the women's movement in Vienna and in other parts of Austria. In its first year, this self‐help group was open to all women who were interested; from winter 1977, the group was advertised as a closed one. Already in the summer of 1976, a self‐help group without thematic focus led by a woman called Traude was announced (AUF No. 7, 1976). The self‐help group “Feminist Therapy,” first announced in winter 1976/77 (AUF No. 9, 1976), probably arose from this initially unspecific self‐help group. In the 10th issue of AUF magazine, which was dedicated to the topics “medicine” and “self‐help,” Traude introduced and described the group in an article called *Feminist Therapy* (p. 27 f). At that time, a total of five women participated (AUF No. 10, 1977, p. 27).

While North American activists had initiated consciousness‐raising as they went along and had somewhat eclectically and often implicitly drawn on Psychological and other Psy‐expert concepts, Viennese activists soon had resources at hand that had traveled to the German‐speaking countries mostly from North America—but noteworthy not from the East Coast, where the radical feminist practice of consciousness‐raising had originated. They also explored Psy‐expert knowledge themselves. Three members of the working group “Feminist Therapy,” including Traude, apparently participated in Gestalt therapy seminars, where they found that the method was suitable for getting to know themselves better and working on themselves (AUF No. 10, 1977, p. 27). The other participants are also presented as having gained experience in Gestalt therapy and bioenergetic seminars. Notably missing are psychoanalytic resources. Apart from the generally strained relationship between feminism and psychoanalysis (see Buhle, 1998), the Austrian post‐war context for psychoanalysis again needs to be taken into account. While during and after WWII psychoanalytic theory flourished in the diaspora, it gained only little traction in German‐speaking academia. In Vienna, for example, psychoanalysis was taught only in therapy training institutions until 1971, when the *Institute for Depth Psychology and Psychotherapy* was founded and affiliated with the Faculty of Medicine at the University of Vienna. In contrast to the Faculty of Medicine, the attitude towards psychoanalysis at the Department of Psychology under Dean Hubert Rohracher was icy. Students of Psychology demanded to be taught about psychoanalysis but not even the offer of additional funds for this “new” field could overturn Rohracher's aversion toward psychoanalysis; the new institute was affiliated to the Faculty of Medicine and not to the Department of Psychology (Plangl, 2004). This example is not an outlier but rather typical for the relations between academic Psychology and psychoanalysis in the German‐speaking countries where the disciplines of psychiatry and psychotherapy (including psychoanalysis) are strongly divided from Psychology. This division between psychotherapy and Psychology, which is even more pronounced than that between psychoanalysis and Psychology, might be another reason for the ironic fact that feminist activists and feminist Psychologists in the German‐speaking countries engaged with psychoanalysis not directly, but through a detour of US American feminist readings of psychoanalysis.

Theoretical resources of the self‐help group “Feminist Therapy” also included *Transactional Analysis* by Eric Berne and readings about Gestalt therapy. Furthermore, the women relied on the few documents available at the time that described therapy approaches specific to women, for example, *Feminism as Therapy*. This book was one of two that were translated to German language soon after their circulation in feminist circles: *Getting clear—Ein Therapiehandbuch für Frauen* (original title: *Getting clear: Body work for women*) by Rush (1973) and *Frauentherapie: Frauenbewegung als heilende Energie* (original title: *Feminism as Therapy*) by Anica Vesel Mander and Anne Kent Rush (1974; German translation 1976).^6^

We infer from the above‐mentioned AUF article on a Viennese feminist therapy group (AUF No. 10, 1977, p. 27) that rather soon after the German translation of *Feminism as Therapy* in 1976, Viennese activists took inspiration from the book by 1977 at the latest. The Viennese feminist therapy group combined “therapy principles” (Rush, 1973) with practices of feminist self‐help groups, which is reflected in the sequence of meetings described by Traude:

We meet on time once a week. At the beginning we take half an hour time for medical self‐examination. Then we begin. We sit on the floor in a circle. We do not smoke, eat or drink. After about an hour we take a break. Then we continue working. We try to make it so that each meeting is led and prepared by a different woman. This does not mean that she is responsible for the evening. She only tries to make the participants [“die Arbeitenden”] aware of what is going on, not to talk anything away or swallow anything. (AUF No. 10, 1977, p. 27 f)

The first half‐hour was devoted to a gynecological self‐examination, which was a common practice in feminist self‐help groups at that time. But Traude firmly stated: “We do not practice self‐help in the medical but in the psychological field. We regularly examine ourselves with the speculum and write down the results, but we do not research anything” (AUF No. 10, 1977, p. 27). In their group sessions, the women wanted to trace their inner impulses, uncover them, and reduce themselves to the “essential.” The activists were concerned with recognizing their own needs to learn to demand that others satisfy them. Traude describes this as particularly difficult for the women: “Most women have never learned to make demands” (AUF No. 10, 1977, p. 28). Another focus was on the analysis of body language. Participants tried to get closer to themselves by observing their own movements and impulses: “Our body language shows a lot of our emotions, only we often do not want to read it or cannot do it anymore” (p. 28). Understanding and feeling the body was promoted with the help of bioenergetic exercises.

The “rules” for women's talking groups suggested by Wagner, and the practice of “self‐help in the psychological field” described by Traude, significantly differ from consciousness‐raising as conceptualized by members of Redstockings (Sarachild, 1970). The notions “talking groups,” “self‐awareness groups,” and “self‐help” already indicate that socio‐political analysis was a minor goal compared to consciousness‐raising groups. In talking groups or self‐awareness groups, women both practiced and aimed at new and more confident modes of communication. Their practices mainly took place on the level of *experience* while in consciousness‐raising, the early, more experience‐based stages of the group practices served as ultimate goal of *analysis* and *political actio*n. In other words, talking groups and self‐awareness groups were rather psychologized and self‐change took center stage as a goal in and of itself. One might call this process *individualization*. Yet, we believe that this notion does not do justice to the fact that the feminists whose relation to Psy‐expert knowledge and to psychologization we have analyzed themselves struggled to avoid individualizing social problems.

## 6 CONCLUSION

In his history of the expansion of Psy‐discourses and practices in Germany, Tändler (2016) has emphasized the hybrid and often contradictory ways in which socio‐political engagement became intertwined with psychological emancipation in Germany in the 1970s. Inspired by the reform spirit of the late 1960s, ideas as well as individuals frequently traveled between the fields of Psychology, counseling, and therapy, and the field of left emancipatory politics. Not only did Psy‐professions politicize and sociologize mental health and schemes of thinking, feeling, and acting, new social and political movements such as left cadre groups, communal groups, but also feminist groups, aimed to link social, individual, and sexual emancipation by incorporating psychological practices of self‐emancipation. As a consequence, members of these groups often considered psychological reflexivity, knowledge, and techniques to be genuine tools of political engagement: The transformation of social and intimate relationships should provide the point of departure of large‐scale social change.

Yet, as Tändler (2016, p. 144f.) notes, the psychologization of political activities and relations was soon criticized for its undifferentiated appreciation of emotions. Importantly for our purposes, he argues that the critique of psychologization itself accelerated the shift in psychological discourses and practice that according to Tändler led to the so‐called “psychoboom” in Germany. Serving as a way of social distinction in an expanding field of psycho‐therapeutic services, the “critique of therapy and therapeutization was part of the background music of the psychoboom more or less from its start” (p. 177). The critique of psychologization itself became a motor that kept the process of psychologization alive.

From the late 1970s onwards, the development of the concept psychologization was intricately connected with a critique of “therapeutic culture” (see Aubry & Travis, 2015a). When Christopher Lasch (1978) launched his now (in)famous critique of therapeutic culture, psychologization was one important, yet also contested element of the historically changing relationship between the women's movement and the Psy‐disciplines. Lasch and “his cohort” (Satter, 2015, p. 120) have been criticized widely—and in our view, rightly so—for misrepresenting or opposing the women's movement (see Aubry & Travis, 2015b; Hochschild, 1983; Lunbeck, 2015; Schmidt, 2020) and for “trivializing the domination of whites over black or of men over women” (Satter, 2015, p. 120). Berryl Satter has proposed to “distinguish between therapeutic culture critics' denigration of the left's legitimate interest in the interplay between the psychological and the political and their insights into the perils of trumpeting the healing power of emotion” (p. 130). De Vos (2013) has conceded Lasch's (1978) “conservative undertow” (p. 80) but concluded that Lasch's critique was mainly directed against the academization of political movements and that he “tried to grasp the dynamics of academization and psychologization” (p. 81). As we have tried to demonstrate, however, academization and individualization are but two dimensions of the multi‐layered process of psychologization. The diffusion of Psy‐expert discourse does not necessarily lead to these processes but can also be employed to analyze the psychological dimensions of oppression, which may aid political action. As Satter (2015, p. 130) has highlighted, feminist consciousness‐raising, in particular, has “showed how attention to personal experience, accompanied by analysis, led to positive transformations in women's lives, from the rewriting of sexual assault laws to the relative opening of job markets.”

In this study, we have analyzed psychologization processes in the US and German‐speaking women's movements that occurred around the practice of consciousness‐raising and its various offspring. We have argued that consciousness‐raising arrived in the German‐speaking countries in an already psychologized fashion. We do not want to suggest a linear development of increasing psychologization as the practice of consciousness‐raising traveled from North America to the German‐speaking countries. However, we think it is rather safe to conclude that consciousness‐raising was increasingly psychologized in its context of origin rather early on and that it reached most other countries in an already psychologized fashion.

At the core of consciousness‐raising was not only a tension between the personal and the political but also the promise to bridge it (see Ruck, 2015). The same can be said for feminist therapy as Mander and Rush (1976) imagined it. This is not to claim that the tensions never erupted into conflicts, however. Quite the opposite is the case: Consciousness‐raising was a contentious practice even within the collectives credited for its inception and popularization. As Echols (2009) has elaborated, some of the most pervasive conflicts in these original collectives centered around the question of whether or not consciousness‐raising privileged self‐transformation over action. The reservations voiced by critics of consciousness‐raising within US women's liberation collectives mirrored the concerns of early consciousness‐raising practitioners against the de‐politicized and individualized versions of the practice that were popularized soon after its inception. These reservations were echoed in the caveat against the privileging of personal change Mander and Rush uttered, and which the Munich Psychologists (also) formulated vis‐à‐vis *Feminism as Therapy*. It seems that with each transformation of the practice of consciousness‐raising and with each new context the practice found itself in, similar conflicts and debates would be rehashed.

We suggest that to understand the psychologization of consciousness‐raising, we need to consider four dimensions that are each characterized by the interrelation between psychologization and its critique: First, psychologization can be framed as the *diffusion of Psy‐expert discourse beyond the borders of the Psy‐disciplines* and into other social domains (see De Vos, 2014; Parker, 2015). We have argued that Sarachild's (1978) lack of citations and of explicit engagements with psychoanalytic or Psychological sources was largely implicit while explicit appropriations of Psychological (mostly psychotherapeutic) concepts can be found in *Feminism as Therapy* and Viennese activists listed not only the published psychotherapeutic und feminist sources they drew on but also their own training in psychotherapeutic methods.

Second, our observations that feminists' references to Psy‐expert discourse were either implicit or explicit points to another dimension of psychologization that relates to the diffusion of Psy‐expert discourse but cannot be equated with it: *academization*. We have identified, for example, Mander and Rush's (1974) explicit engagement with psychoanalytic or Psychological authors as signs of academization. What is more, we have proposed that Mander and Rush pushed forward the process of academization even though they explicitly criticized and denounced academic practices. Indeed, one might push the argument even further and claim that decrying academic practices can itself be a marker of academization. Note, however, that academization is not the same as the spreading of Psy‐expert discourse into other social fields, as academization may also entail habitual academic knowledge‐related practices such as acknowledging the validity of knowledge claims only when they refer to the principle of scientific “facts” or the dichotomy between “true” and “untrue” or practices that are not necessarily unique to but part of the academia such as citing sources, defining key terms, or engaging with primary sources. This is the aspect of academization we see at work increasingly as the practice of consciousness‐raising was taken up and analyzed in scientific publications on psychotherapy (Brodsky, 1973) and when Psychologists started to study the effects of consciousness‐raising using canonical Psychological methods (e.g., Follingstad et al., 1977).

A third aspect came into place as consciousness‐raising itself was increasingly academized and infused with Psy‐expert discourse: *individualization*. Psychologization as individualization happened rather early on as women would increasingly used consciousness‐raising to enhance their well‐being and not progress to the more political and analytical steps of the process. Though this development was criticized by radical feminists early on (see Echols, 2009), individual well‐being continued to be a part of consciousness‐raising. This holds especially true for remnants of consciousness‐raising in feminist therapies. In the case of the German translation of *Feminism as Therapy*, we have pointed out that the practices suggested for “talking‐groups” were mainly located on the level of experience, while consciousness‐raising also entailed analysis and political action.

We have argued, however, that the notion of individualization fails to acknowledge that all feminist P/psychologies we have analyzed in this study struggled to avoid the pitfalls of psychologization, that they themselves criticized processes of individualization and academization, and that they strove to overcome them. This brings us to a fourth dimension of psychologization that we believe to be especially vital for our case studies: *meta‐psychologization*. Jan de Vos has defined meta‐psychologization as a “psychology of psychologization” (De Vos, 2013, p. 3) and has warned that a critique of psychologization might itself turn into a psychology, which itself may need to be criticized for its psychologizing tendencies, if it fails to address the socio‐structural and political dynamics of the dispersion of psychological knowledge and practices into different social fields. Somewhat in contrast to de Vos' proposal, we employ meta‐psychologization as an analytical term that tries to understand the interrelations between psychologization and its critique (see Balz & Malich, 2020; Malich & Balz, 2020, for a similar endeavor). It is the notion of meta‐psychologization that allows us to finally tie our analyses together with the historiographic distinction between “little p” psychology and “big P” Psychology (Pickren & Rutherford, 2010, p. xix; Richards, 2010) and to better understand the interrelation between feminist psychologies produced in the social field of feminist activism and the Psy‐disciplines. More specifically, it serves to grasp the kind of psychologization at play in the way Viennese feminist activists practiced self‐awareness: These feminist psychologies relied on a psychological practice that was itself a result of a critique of Psychology—but a psychology nevertheless; it was a form of consciousness‐raising that had already spiraled through the bottleneck of psychologization.

## CONFLICT OF INTERESTS

The authors declare no conflict of interests.

## Data Availability

No data are associated with this study.

## ENDNOTES

^1^
The translation of the title is all the more remarkable as the rest of the translation stays rather close to the English original and “Feminismus als Therapie” would be a feasible translation.

^2^
The heading “Freud was confused” is one of the examples where the German translation deviated from the English original, the German translation being “Freud irrte sich” (Freud was wrong).

^3^
In the case of publications that were originally published in German, we refer to the German original. All translations of German originals have been made by the authors.

^4^
Volkshochschulen are schools of continued adult education similar to community colleges. They do, however, not grant academic certificates.

^5^
The questions included in this questionnaire, as listed by Geiger and Hacker (1989), are virtually identical to the list of questions for “women's talking groups” suggested in the German translation of the book Frauentherapie (Feminism as Therapy). These questions are not part of the English original but were added to the German translation in a chapter by Angelika Wagner, a member of the Munich group of Psychologists who initiated the translation of Feminism as Therapy.

^6^
In the following, we refer to the German translations except for direct quotes, which are cited from the English original.
